# Unimanual Intensive Therapy with or without Unaffected Hand Containment in Children with Hemiplegia. A Randomized Controlled Pilot Study

**DOI:** 10.3390/jcm9092992

**Published:** 2020-09-16

**Authors:** Rocío Palomo-Carrión, Elena Pinero-Pinto, Sara Ando-LaFuente, Asunción Ferri-Morales, Elisabeth Bravo-Esteban, Helena Romay-Barrero

**Affiliations:** 1 Department of Nursery, Physiotherapy and Occupational Therapy, Faculty of Physiotherapy and Nursery, University of Castilla-La Mancha, 45071 Toledo, Spain; Rocio.Palomo@uclm.es (R.P.-C.); Asuncion.Ferri@uclm.es (A.F.-M.); Elisabeth.Bravo@uclm.es (E.B.-E.); Helena.Romay@uclm.es (H.R.-B.); 2 Physiotherapy Research Group in Toledo, GIFTO, 45071 Toledo, Spain; 3 Department of Physical Therapy, Faculty of Nursery, Physiotherapy and Podiatry, University of Seville, 49001 Seville, Spain

**Keywords:** containment, family, home, infantile hemiplegia, intensive therapy, modified constraint induced movement therapy, rehabilitation, unimanual therapy without containment, upper extremity

## Abstract

Children with hemiplegia have lower spontaneous use and quality of movement in the affected upper limb. The modified constraint-induced movement therapy (mCIMT) is applied to improve the affected upper limb function. The objective of this study was to study the efficacy of unaffected hand containment to obtain changes in the function of the affected upper limb after applying two unimanual therapies. A randomized controlled pilot study was performed with 16 children diagnosed with congenital infantile hemiplegia, with eight children randomized in each group (average age: 5.54 years; SD: 1.55). mCIMT and unimanual therapy without containment (UTWC) were applied, with a total of 50 h distributed in five weeks (two h/per day). Two assessments were performed (pre- and post-treatment) to evaluate the affected upper limb spontaneous use, measured with the Shiners Hospital Upper Extremity Evaluation (SHUEE), and the quality of movement, measured with the Quality of Upper Extremity Skills Test (QUEST scale). The progression of the variables was different in both groups. The results are expressed in the median of the improvement percent and interquartile range (IQR). The spontaneous use analysis showed an improvement percent of 31.65 (IQR: 2.33, 110.42) in the mCIMT group with respect to 0.00 (IQR: 0.00, 0.00) in the UTWC group. The quality of movement increased in the mCIMT and UTWC groups, 24.21 (IQR: 13.44, 50.39), 1.34 (IQR: 0.00, 4.75), respectively and the greatest increase was obtained in the grasp variable for both groups. The use of unaffected hand containment in mCIMT would produce improvements in the affected upper limb functionality in children with hemiplegia (4–8 years old) compared to the same protocol without containment (UTWC).

## 1. Introduction

Infantile cerebral palsy (ICP) is a non-progressive encephalopathy that produces a series of permanent disorders, affecting motor and postural development in children [1]. The disease prevalence in developed countries is 2–2.5 cases per 1000 live births [1]. One of the most frequently occurring forms of ICP is hemiplegia, where one vertical body side is affected, as a consequence of brain damage that primarily affects one hemisphere [2]. Movements in the affected upper limb are slower and clumsy and accompanied by mirror movements. Moreover, there are deficits in the selective control in the fingers of the affected hand. Consequently, there is a reduction in the use of the affected hand, commonly known as “developmental disregard”, which interferes with activities of daily living [3]. Children with hemiplegia do not acquire a typical movement experience in their affected upper limb, unlike adults who have suffered a stroke later in their lifetime. Thus, the therapy used must provide the opportunity to experiment with the affected side, granting as much functionality as possible to the affected upper limb [4].

The time between the ages of three and 10 years is critical for motor control development in children, as evinced by behavioral [5,6,7] and neuroimaging studies [8,9]. Within this age range, motor and sensory areas develop first, followed by higher order areas, such as the prefrontal cortex, which develop later [9]. In the study by Abd El-Kafy et al. [10], children with an age range between four and eight years were recruited, due to the repertoire of movements that allows the development of efficient motor solutions to environmental restrictions. Therefore, interventions carried out at these ages are focused on increasing practice in the affected upper limb. Many adolescents are motivated to improve motor function through being aware of their deficits, thus they are keen to improve for the sake of social inclusion [10]. This contrasts with young children, whose motivation and participation in therapy is usually driven by their parents or caregivers [10].

Constraint-induced movement therapy (CIMT) is designed to improve the affected upper limb motor function after stroke, and consists of three key components: (1) repetitive, unimanual task-oriented training for six consecutive hours per day during 10–12 days; (2) adherence-enhancing behavioral strategies (transfer package); and (3) constraining the use of the less affected arm, usually by wearing a glove during waking hours [11,12]. CIMT modifications were proposed by Page et al. [13,14] using less than three non-consecutive hours of therapy per day applying the unaffected arm containment. These studies reported an increased use in the affected arm [13,14].

Interventions with modified CIMT (mCIMT) [13,14] at an early age could expand primary neural networks through the experience and practice of their affected upper limb, since it implies a structured practice, demands attention to the task and encourages the practice and use of the affected segment. Thus, the training and repetitive tasks would be aimed at treating children from four years of age, who have the ability to execute the task for longer periods of time [13].

The mCIMT is effective at promoting the functional use of the affected upper limb in children with hemiplegia [15,16,17,18]. Different studies in stroke patients have shown that mCIMT improves performance in tasks such as picking up a cup, grasping a spoon or holding a book [13,14,19,20]. The home environment provides a rich natural context to facilitate motivation, engagement and repetition in functional activities of daily living [21,22]. A “transfer package” technique facilitates treatment gains into real-world activities, such as reinforcement of treatment adherence and the emergence of new behaviors, thereby improving the spontaneous use of the trained affected upper limb in infantile hemiplegia [23,24]. Thus, mCIMT improves functionality in the affected upper limb through enhanced practice, which could be induced by the unaffected hand containment [23].

It is unknown whether the benefits of mCIMT result from the repetition of activities or the use of unaffected hand containment. Therefore, this study assessed the effects of two unimanual therapies with the same dose and activities for the affected upper limb: mCIMT (with unaffected hand containment) and unimanual therapy without containment (UTWC) in children with hemiplegia. Our hypothesis proposes that children with hemiplegia who complete the mCIMT would have a higher increase in affected upper limb functionality than children who complete the UTWC. The objective was to assess the efficacy of unaffected hand containment to increase the spontaneous use and quality of movement in the affected upper limb in children with hemiplegia between four and eight years old.

## 2. Materials and Methods

The study was approved (060-13) by the San Pablo CEU University ethics committee of Madrid (Spain) according to the World Medical Association Declaration of Helsinki. Before the study began, the consent of the families and children’s caregivers was provided.

### 2.1. Study Design

A simple-blind (evaluator), randomized, controlled pilot study was designed (clinical.gov registry number: NCT02178371) using two parallel intervention groups. The participants were recruited through convenience sampling, and they were randomized using Epidat v.4.2 software.

### 2.2. Participants

The study sample was recruited from the HEMIWEB association (The association of infantile hemiplegia in Spain), following the establishing of inclusion and exclusion criteria. The inclusion criteria were the following: congenital infantile hemiplegia, aged between 4 and 8 years, lack of use of the affected upper limb, exceeding 10° of extension of metacarpophalangeal and interphalangeal joints and completing 10° of active extension of the wrist joint, adequate cognitive development to understand verbal orders for the proposed tasks, and cooperation in their execution [25,26,27,28,29]. The exclusion criteria were defined as the following: visual problems, significant balance disturbances that prevent performing the tasks, diseases not related to hemiplegia, pharmacoresistant epilepsy and botulinum toxin infiltration within 3 months prior to the intervention.

### 2.3. Procedures and Interventions

The planning of the project and its dissemination to recruit the sample were carried out from January 2015 to December 2017. In the period of January 2018 to December 2018, the participants were selected and the meetings with the families were held. Assessments of the baseline situation (pre-treatment) of the children were conducted in January–October 2019. The interventions were carried out between January and October–November 2019, and the assessments of the final situation (post-treatment) were conducted between February and December 2019.

An informational meeting was held with all families, in which they signed an informed consent. Then, they were trained in the execution of each therapy (mCIMT: experimental group and UTWC: control group) by the therapist, teaching them how they should carry out the activities at home in each intervention group. The treatment was only initiated when the families and children were confident about it. The family and therapist met every week to assess the activities and make adjustments if necessary. A weekly follow-up was implemented to avoid any complications and increase the treatment adherence. The follow-up with the families was conducted online, reviewing all the activities and modifying those that were too difficult for the child, maintaining great therapist-family-child feedback. This concept means that the family is a key component of the child’s environment and the relationship with the therapist (through follow-up) can be used as the context to deliver critical components (i.e., intensity, repetition, feedback) of established therapies with the child [30]. Hadders-Algra et al. [31] state that a family-centered approach creates a richer and more varied array of opportunities by coaching the family to encourage the child to use the affected upper limb in the usual environment.

The proposed objective between the therapist and the family was to increase the spontaneous use of the affected hand, that is, that the affected hand could assist in the execution of bimanual activities of daily living: eating, hygiene, dressing-undressing, etc. Therapist-family-child feedback was maintained within a weekly family-centered program, where the monitoring table, the activities, the doubts of the families, the complications, the manifestations of the children about the activities or changes to promote their motivation following their preferences were reviewed. In this way, adherence to the treatment, continuity and compliance with the proposed dose and activities were promoted.

**Figure 1:**
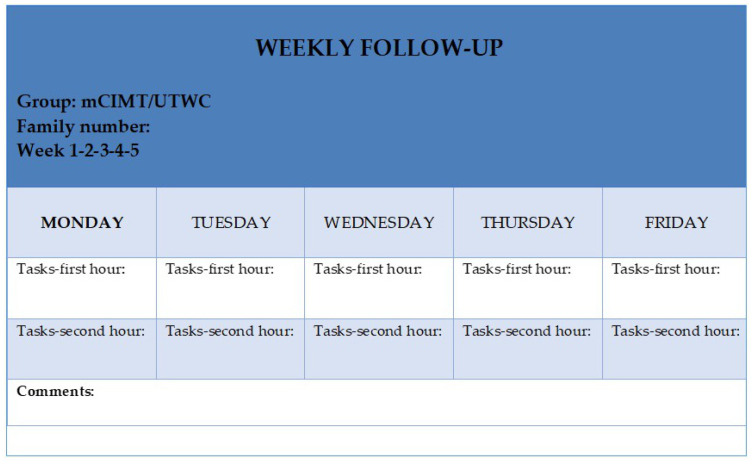
Weekly follow-up for families.

The families were requested to fill in a table with the execution time of each activity, the different activities the child performed in the first and second hour and the child’s behavior towards it (Figure 1).

**Figure 2:**
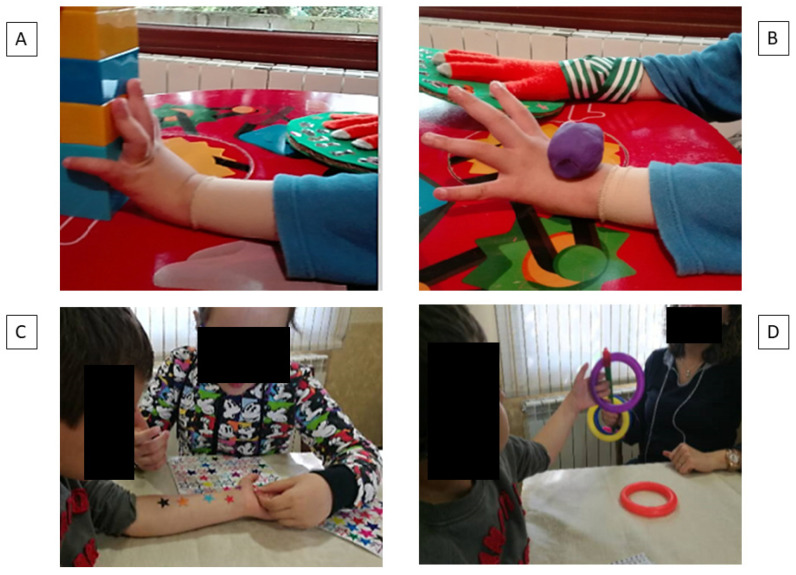
Activities in both protocols. The (**A**,**B**) pictures show two activities performed with modified constraint-induced movement therapy (mCIMT), using the containment in the unaffected hand (right hand) to work the wrist extension in the affected hand (left hand). The (**C**,**D**) pictures show two activities performed with unimanual therapy without containment (UTWC). The child works the supination movement in picture C, where the mother puts stickers on his forearm, and, in picture D, he removes rings towards supination.

Two 5-week intensive protocols of mCIMT or UTWC were executed at home. The tasks’ difficulty gradient for the affected upper limb was programmed for 2 h per day from Monday to Friday with a total therapeutic dose of 50 h [13,14]. The children were requested to perform the structured activities for two non-consecutive hours, separated by at least 30 min of rest. The families were advised to set aside one hour in the early afternoon and another hour in the late afternoon to ensure that the child was attentive, frustration-free and effort-tolerant. The families were also instructed to run a full hour, repeating the activity and designing a story, in which the child was the protagonist and the activity was an enjoyable game to complete. The unaffected hand was partially contained in the mCIMT group and free in the UTWC group. Each family built the partial containment as a glove with a rigid cardboard base to prevent mirror movements, leaving the wrist joint free (Figure 2).

Both therapies were carried out by the children’s families in their usual environment (home) to encourage the learning of the affected upper limb in activities of daily living. In addition, the activities were created with a unimanual component in a general way based on the age and the affected upper limb limitations of the children included in the baseline restrictions of movement, as well as on the interests of the children (Appendix A Table A1). The activities were the same for both groups (the same activities, dose, guidelines…), with the difference of the containment in the unaffected hand for the mCIMT group. The activities were programmed to work different movements that were limited in the affected upper limb: shoulder flexion, elbow extension, supination forearm, wrist extension and grasp. Each activity was repeated for around 10 min to obtain a learning about a functional strategy to use in their usual activities.

### 2.4. Outcome Measures

Two assessments were performed. The first assessment was focused on obtaining the data before the treatment, i.e., in week 0 (baseline situation, immediately before starting treatment), whereas the second assessment was conducted at the end of the treatment, i.e., in week 5 (a total dose of 50 h).

#### 2.4.1. Primary Measures

The spontaneous use analysis (SUA) was carried out through nine proposed activities included in the modified House score scale, with a score of 0 when there is no activity of the affected upper limb and a score of 5 when there is total participation of the affected upper limb in the bimanual task proposed by the test. A total score of 45 points represented 100% spontaneous use.

The dynamic positional analysis (DPA), conducted in 16 activities, assesses the alignment of the upper limb when performing the task, with a maximum score of 72 points, whereas the grasp–release (GR) action evaluates the children’s ability to close and open their fingers with the wrist held in three positions: flexion, neutral and extension (6 points is the maximum score). The variables were measured using the Shriners Hospital Upper Extremity Evaluation (SHUEE) [32], which has been validated for children with hemiplegia (3–18 years).

The absolute mean differences between the two scoring sessions for three raters were 1.2 and 1.0 for SUA and DPA, respectively. There was excellent intra-observer reliability between the two sessions with regard to both SUA (r = 0.99) and DPA (r = 0.98). The assessment of inter-observer reliability revealed absolute mean differences between four raters of 3.8 (SD: 2.4) and 3.7 (SD: 2.6) for SUA and DPA, respectively. These differences were significantly different from 0 (*p* < 0.001); however, the magnitudes of these differences were not important with regard to total score or clinical interpretation [32]. There was excellent inter-observer reliability for both SUA (r = 0.90) and DPA (r = 0.89) [32]. There was 100% agreement within and between examiners for GR. The Shriners Hospital Upper Extremity Evaluation for Children showed a fair correlation with the self-care scaled score from the Pediatric Evaluation of Disability Inventory (r = 0.47) and a good inverse correlation with the non-dominant total time section of the Jebson–Taylor test (r = −0.76) [32].

#### 2.4.2. Secondary Measures

The movement quality (MQ) was measured using the quality upper extremity skills test (QUEST) [33], validated for ICP (18 months to 8 years of age). The test consists of 36 items, divided into four categories: dissociated movements (DM), which is the capacity to perform a specific movement using a joint; grasp (G), which is referred to what type of grasp the child uses for small objects, how the child holds a pencil and whether there are atypical or incorrect positions during the activity; weight bearing (WB), which is the correct position of the joints in the upper limbs to lean on a surface using the hands; and protective extension (PE), which is the position that the upper limbs adopt to counteract an imbalance. Each category can be scored from 1 to 100 points, and the result is expressed in percentages (%).

Inter-rater reliability was reported to be excellent for total score from 0.90 to 0.96. Test-retest reliability was high for total score at 0.95 and in the domains ranged from 0.75 (protective extension) to 0.95 (dissociated movements) [33,34]. Construct validity was assessed by correlating QUEST total score with the therapist’s ratings of left- and right-hand function on an 11-point scale and with chronological age. Correlations with hand function ratings were reported to be high for the left hand (0.72) and moderate (0.58) for the right hand [33,34]. The correlation between QUEST total score and chronological age was low (0.33). The response to clinical change was not sufficiently studied, thus a change of 10 points in the QUEST total score can be considered as a great improvement [34].

### 2.5. Statistical Analysis

The statistical analysis of the data was performed using SPSS v20.0 for Windows (SPSS Inc., Chicago, IL, USA). Given the small sample size, non-parametric analyses were used with the Mann–Whitney U-test to determine the inter-group differences for the variables, and the percent of improvement for each subject was calculated. The Wilcoxon test for paired samples was performed to compare before–after treatment results in the same group for both therapies. Fisher’s exact test was used to determine inter-group differences according to sex. The results are shown as the median and interquartile range (IQR) with a confidence interval of 95%. All those values with *p* value < 0.05 were considered statistically significant.

## 3. Results

**Figure 3:**
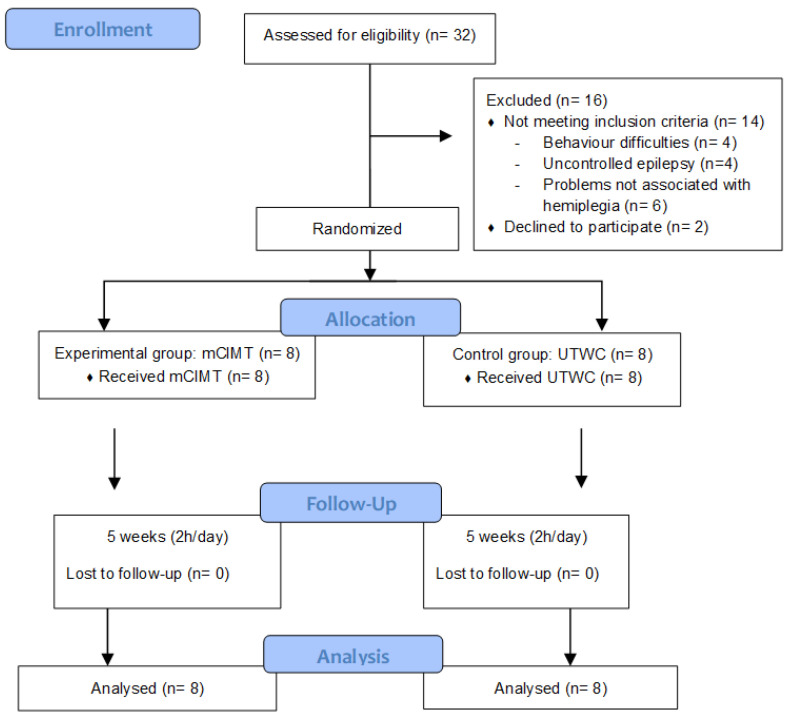
Consort flowchart. Allocation of the sample, therapies performed and analysis.

A total of 32 subjects were recruited, of which 14 were excluded for not meeting the inclusion criteria and another two eventually decided not to participate. The remaining 16 subjects met the inclusion criteria established and were randomly allocated in either of the two intervention groups. Eight children were included in the mCIMT group and the other eight children in the UTWC group (Figure 3).

Fifty percent of the participants were males and the other 50% were females and 100% of them were diagnosed with congenital hemiplegia (perinatal stroke). Of the entire sample, 62.50% had left hemiplegia, with the involvement of the right half of the body being less representative (37.50%), and 56.25% were classified as Manual Ability Classification System (MACS) level II. All the children had infantile hemiplegia due to perinatal stroke (Table 1).

The ages were between four and eight years, with an average of 5.54 years (SD: 1.55 yr). There were no statistically significant differences between groups for age (*p* = 0.78) and sex (*p* = 1.00).

All the parents completed the weekly follow-up (Figure 1), recording the activities carried out and the time dedicated to each activity until completing the 2 h of daily treatment. In addition, when the hours were not completed, it was also recorded, in order to calculate the total treatment dose. The mean total dose recorded for the mCIMT and UWTC groups was 47 h and 45 h and 30 min, respectively.

### 3.1. Primary Results

There were no statistically significant inter-group differences in the baseline assessment (week zero) for SUA, DPA or GR according to the Mann–Whitney U-test (*p* > 0.05). On the other hand, there were statistically significant inter-group differences after treatment (week five) for SUA and DPA (*p* < 0.05), but not for GR (*p* = 0.08), according to the Mann–Whitney U-test. All the variables measured with the SHUEE evaluation obtained significant differences in the percent of improvement (*p*-value < 0.01). These differences were present in the mCIMT group, with respect to the UWTC group, since SUA showed an improvement percent of 31.65 (IQR: 2.33, 110.42) in the mCIMT group with respect to 0.00 (IQR: 0.00, 0.00) in the UTWC group. For DPA, an improvement percent of 15.17 (IQR: 4.77, 78.36) was obtained in the mCIMT group compared to 0.00 (IQR: 0.00, 0.00) in the UTWC group. Lastly, GR obtained an improvement percent of 41.67 (IQR: 0.00, 100.00) compared to 0.00 (IQR:0.00, 0.00) in the UTWC group. Thus, the percentages of improvement were stable and unchanged for the three variables in the control group (Table 2). Figure S1 shows the Percent of improvement Shuee evaluation variables (supplementary materials).

### 3.2. Secondary Results

There were no statistically significant inter-group differences in the baseline assessment (week zero) for the different variables according to the Mann–Whitney U-test (*p* > 0.05). On the other hand, there were statistically significant inter-group differences for all variables after treatment (week five) according to the Mann–Whitney U-test (*p* < 0.05). All the variables measured with the QUEST scale obtained significant differences in the percent of improvement with a *p* value ≤ 0.01. These differences were present in the mCIMT group, with respect to the UWTC group, since the MQ showed an improvement percent of 24.21 (IQR: 13.44, 50.39) in the mCIMT group with respect to 1.34 (IQR: 0.00, 4.75) in the UTWC group. For DM, an improvement percent of 38.68 (IQR: 9.42, 81.24) was obtained in the mCIMT group compared to 1.40 (IQR: 0.00, 7.77) in the UTWC group. GR showed an improvement percent of 38.92 (IQR: 8.35, 156.78) in the mCIMT group compared to 4.67 (IQR: 0.00, 42.68) in the UTWC group. Lastly, the improvement percentages for WB and PE were higher in the mCIMT group, with 12.68 (IQR: 2.04, 36.11) and 17.25 (IQR: 0.00, 25.92), respectively, with respect to the UTWC group, where the values of improvement percentages for the same variables were 0.00 (IQR: 0.00, 0.00). (Table 3). Figure S2 shows the Percent of improvement QUEST scale variables (supplementary materials).

## 4. Discussion

The effect of different therapeutic strategies in children with hemiplegia might be influenced by two characteristics: “poor or non-use” of affected upper limb and mirror movements [35]. This suggests that the containment of the unaffected hand could reduce the mirror movements that occur in infantile hemiplegia, thus influencing the execution of uni and bimanual activities [36]. It also suggests that having the unaffected hand free, with no specific role in the activity, would not influence the mirror movements. Therefore, some of the studied variables (SUA, DPA, GR, WB and PE) did not vary in the UTWC group, since children did not obtain variability in motor behavior, change of strategies or quality of movement due to the presence of the free unaffected hand without a specific role.

The improvements obtained in dissociated movements and grasp–release in the mCIMT group and not in the UTWC group became visible in the affected upper limb alignment in motion when the task was executed, assessed through dynamic positional analysis and a functional grasp test with different wrist joint positions. The quality of movement in the affected upper limb improved, and these changes were observed in the SHUEE evaluation for the mCIMT group, not only for the dynamic positional analysis and grasp–release action, but also in spontaneous use, which is a very important concept, since the non-use of the affected upper limb influences the non-participation in activities of daily living, thus potentially producing frustration [37]. Comparing the data between the mCIMT and UTWC groups, it was observed that the mCIMT group showed improvements in the affected upper limb function. This could be due not only to the repetition of unimanual tasks and the strategy opportunities provided, but also to the use of unaffected hand containment. Consequently, when the unaffected hand was contained, the affected upper limb spontaneous use was faster [13,14]. In the UTWC group, there was no involvement of the unaffected hand in the tasks, although the child was free to move it, with the possibility of using it to try and do the task; thus, the parents had to remind the child not to use the unaffected hand during the treatment activities. In this situation, the brain may be obtaining information from both upper limbs and this would produce less affected limb participation than if the unaffected hand was contained [13,14]. This would explain the absence of change in the results of improvement percent for the UTWC group, since the children did not have the same chance to practice with the affected upper limb due to the presence of verbal or physical reinforcement from the parents not to use the unaffected hand. In the mCIMT group, spontaneous use, measured with the SHUEE scale, showed a significant improvement in the affected upper limb, due to the fact that the children obtained more active participation for the bimanual tasks (proposed in the test). This suggests that the use of unaffected containment in the mCIMT group would improve the affected upper limb perception, and that children were more aware of its existence, perhaps due to cortical changes in the brain after applying this protocol [13]. Thus, having the unaffected hand free, despite the fact that it did not participate in the tasks, may induce lower changes at the brain level, reducing the affected upper limb perception, influencing the beginning of spontaneous use and decreasing the automatic unimanual and bimanual use, since such changes were not obtained for spontaneous use in the UTWC group after five weeks of therapy [16].

The deterioration in the functionality of the affected hand causes limitations in the performance of activities of daily living in children with hemiplegia [38], with alterations in functionality compared to the unaffected upper limb. These alterations manifest as slow and/or discontinuous movements, variability in hand trajectories while reaching for an object, with trunk compensations, and inadequate grasp force [39]. They occur at the structural and functional level, due to the fact that movement deficits reduce the quality of movement of the affected upper limb. Thus, once both unimanual therapy protocols were used, it was observed that, despite using the same dosage (50 h), significant changes with improvements were obtained for the movement quality total score in the mCIMT group and not in the UTWC group. These changes result from the large increase in the improvement of dissociated movements and grasp–release, possibly acquiring an improvement in the selectivity of the use of the joints, which might suggest greater ease in the individualized movement of the fingers; this, in turn, may have facilitated the acquisition of a more precise and fine grasp, which translated into an improvement in trunk movement, since the existing pre-intervention compensations were reduced (head lateralization and flexion and trunk lateralization). In the UTWC group, the greatest increase in the movement quality variables also occurred in the grasp, although not clinically relevant changes, since after therapy a more functional grasp was not observed in the children. Unlike the control group, in the mCIMT group, the parents stated that, after the treatment, the children were able to use the affected hand to assist their actions, holding the object with grasp to manipulate it with the unaffected hand. The reduction in compensations in posture and a more effective grasp in the mCIMT group could influence a higher quality in the reach and grasp of the object, facilitating the participation of the affected upper limb, since these concepts and the action anticipation phenomena are altered in children diagnosed with hemiplegia [40]. Thus, wearing the containment on the unaffected hand would allow a more fluid movement trajectory due to the improvements observed in the variables of the quality of movement.

Comparing the results of movement quality with those reported in the study of Choudhary et al. [18], performed in children with hemiplegia from three to eight years old, a greater increase in total score was observed in the mCIMT group (as in the present study) with respect to the control group, probably as a result of the use of unaffected hand containment. The gains were greater in our study than in the study of Choudhary et al. [18] for the mCIMT group. This could be due to the fact that Choudhary et al. [18] applied a lower dose (20 h) with respect to the present study (50 h), suggesting that the dose is an important factor to consider in the intensive treatment of children with hemiplegia (4–8 years). In both studies, the greatest gains were obtained for the grasp–release subscale in the mCIMT group, being greater in our study, probably due to the longer dose (50 h). The dissociated movements obtained a great increase in the present study (30.46%) for the mCIMT group and a very low increase for the mCIMT group in the study of Choudhary et al. (8%), which could be due to the fact that the children in our study had a low score in the baseline situation (59.38%) in this variable compared to the mCIMT group in the study of Choudhary et al. (75.5%). The baseline situation could influence the final results. This suggests that children with more affectation and a greater dose (30 extra hours) could increase the improvements in these subcategories (dissociated movements and grasp). The use of unaffected hand containment in the mCIMT group improved dissociated movements and grasp in the quality of movement measured with QUEST, which could improve the dynamic positional analysis during the task movement. Therefore, it would be important to obtain a better functional grasp, which would allow for better assistance with the affected hand when the children perform bimanual tasks, resulting in an increase in affected upper limb spontaneous use and, thus, greater participation of the children with their environment.

It is important to highlight that there were no dropouts from the therapies carried out in the study, for any of the groups (mCIMT and UTWC), and that all families completed the activities adequately, without complications. Moreover, there was great coordination between the therapist and the families during the weekly follow-up, which helped them to complete a great treatment dose in both groups, and no important modifications were made to the structured activities during the on-line weekly follow-up sessions, only changes for a different object or one that the family had at home. The children themselves were very participative. This suggests that weekly follow-up and the involvement of the families and therapist allows the family to get involved in the therapy, which motivates the child to perform it, preventing them from abandoning the treatment. All the families commented that the execution within the natural environment allowed them to distribute the therapy time adequately, without stress, since the protocol did not interfere with their needs, which could also benefit the adherence to both treatments. The execution of the high-dose therapy in both groups, the adherence of the family and child to the treatment, the active participation and the absence of parental stress are phenomena that show that the family-centered model is functional, due to the empowerment of the parents through the therapist, and that it allows the children to experiment and participate in their own environment, leading to greater learning opportunities [31]. The stability of parental stress throughout the intervention would suggest that this may have been an effective strategy for avoiding disruption of the psychosocial family dynamics. In the study of Ferre et al. [41], caregivers who applied 90 h of bimanual intensive therapy at home with their children (diagnosed with hemiplegia) showed parental stress levels that were similar to those of caregivers of typically developing children. Therefore, in our study, it can be assumed that the levels of parental stress derived from the intensive treatment may have been adequately solved with the empowerment of the family, the support provided and the follow-up sessions, thus ensuring their adherence to the treatment.

Regarding the limitations of the study, it is important to highlight, firstly, the small sample size, which is why it is considered as a pilot study, and secondly, the absence of a long-term follow-up to assess the maintenance of the results in both groups. Future studies should consider using a larger sample, multicenter trials should be performed to improve the research viability and reliability and follow-up results at six months or longer. Moreover, further research could assess affected upper limb functionality (including a mirror movement assessment) over time and add a bimanual group to compare the changes using both hands without containment and only the affected hand without containment in the UTWC compared with the mCIMT group.

## 5. Conclusions

The use of unaffected hand containment in mCIMT would produce improvements in the affected upper limb functionality in children with hemiplegia (4–8 years old) compared to the same protocol without containment (UTWC). Unaffected hand containment could reduce the “developmental disregard or non-use”, thus increasing spontaneous use and quality of movement in the affected upper limb in children with hemiplegia.

## Figures and Tables

**Table 1:** Baseline measurements per group and total participants.

VARIABLES	Total (n = 16)	mCIMT (n = 8)	UTWC (n = 8)	*p*-Value
**AGE, years (SD)**	5.54 (1.55)	5.63 (1.21)	5.50 (1.12)	0.78
**SEX**				
**Male, n. (%)**	8 (50)	4 (50)	4 (50)	1.00
**Female, n. (%)**	8 (50)	4 (50)	4 (50)	
**HEMIPLEGIA**				
**Left, n. (%)**	10 (62.50)	5 (62.50)	5 (62.50)	-
**Right, n. (%)**	6 (37.50)	3 (37.50)	3 (37.50)	
**MACS score (I–V)**				
**II, n. (%)**	9 (56.25)	6 (75.00)	3 (37.50)	-
**III, n. (%)**	7 (43.75)	2 (25.00)	5 (62.50)	
**SHUEE Evaluation % median. (IQR)**				
**Spontaneous Use Analysis**	64.33 (42.22, 95.55)	70.00 (42.22, 95.55)	63.72 (44.44, 88.44)	0.64
**Dynamic Positional Analysis**	76.39 (45.83, 88.89)	77.78 (45.83, 88.89)	74.39 (48.00, 78.88)	1.20
**Grasp and Release**	64 (50.00, 100.00)	58.34 (50.00, 100.00)	64 (50.00, 100.00)	1.48
**QUEST Scale % median. (IQR)**				
**Movement Quality Total score**	74.15 (56.24, 85,14)	74.15 (56.24, 85.14)	74.15 (60.00, 83.17)	1.76
**Dissociated Movements**	57.82 (49.45, 85.94)	59.38 (50.00, 82.82)	57.82 (49.45, 85.94)	1.92
**Grasp**	66.67 (25.96, 88.88)	62.97 (25.96, 88.88)	66.67 (25.16, 85.18)	1.92
**Weight Bearing**	77.78 (37.04, 100.00)	87 (72.00, 98.00)	94 (76.00, 98.00)	0.44
**Protective Extension**	80.56 (75.00, 100.00)	80.56 (75.00, 100.00)	80 (75.00, 90.00)	1.16

Experimental group: mCIMT: modified constraint-induced movement therapy; control group: UTWC: unimanual therapy without containment. F: female; M: male. MACS: Manual Ability Classification System. Statistical significance when *p* value is < 0.05 (α correction in Mann–Whitney U-test).

**Table 2:** Results and percent of improvement per group and total sample in the variables measured with the Shiners Hospital Upper Extremity Evaluation (SHUEE) evaluation.

SHUEE Evaluation	Total Sample (n = 16)	m-CIMT (n = 8)	UTWC (n = 8)	*p*-Value
**SUA** **Week 0** **Week 5** **Percent of improvement**	64.33 (42.22, 95.55) 84.44 (44.44, 97.78) 34.77 (0.00, 110.42)	70 (42.22, 95.55) 88.87 (84.44, 97.78) 31.65 (2.33, 110.42)	63.72 (44.44, 88.44) 63.72 (44.44, 88.44) 0.00 (0.00, 0.00)	0.64 <0.001 <0.001
**DPA** **Week 0** **Week 5** **Percent of improvement**	76.39 (45.83, 88.89) 79.71 (48.00, 97.22) 2.38 (0.00, 78,36)	77.78 (45.83, 88.89) 88.20 (65.27, 97.22) 15.17 (4.77, 78.36)	74.39 (48.00, 78.88) 74.40 (48.00, 78.88) 0.00 (0.00, 0.00)	1.20 <0.001 <0.001
**GR** **Week 0** **Week 5** **Percent of improvement**	64 (50.00, 100.00) 83.33 (50.00, 100.00) 0.00 (0.00, 100.00)	58.34 (50.00,100.00) 91.67 (66.67, 100.00) 41.67 (0.00, 100.00)	64 (50.00,100.00) 64.40 (50.00, 100.00) 0.00 (0.00, 0.00)	1.48 0.08 0.008

Experimental group: mCIMT: modified constraint-induced movement therapy; control group: UTWC: unimanual therapy without containment. SUA: spontaneous use analysis; DPA: dynamic positional analysis; GR: grasp and release. Results expressed in medians (IQR, interquartile range: Q1, Q3). Statistical significance when *p* value is < 0.05 (α correction in Mann–Whitney U-test).

**Table 3:** Results and percent of improvement per group and total sample in the variables measured with the Quality of Upper Extremity Skills Test (QUEST) scale.

QUEST Scale	Total Sample (n = 16)	m-CIMT (n = 8)	UTWC (n = 8)	*p* Value
**MQ** **Week 0** **Week 5** **Percent of improvement**	74.15 (56.24, 85,14) 83.17 (60.00, 96.66) 9.09 (0.00, 50.39)	74.15 (56.24, 85.14) 94.06 (96.66, 83.11) 24.21 (13.44, 50.39)	74.15 (60.00, 83.17) 75.92 (60.00, 83.17) 1.34 (0.00, 4.75)	1.76 <0.001 0.002
**DM** **Week 0** **Week 5** **Percent of improvement**	57.82 (49.45, 85.94) 83.60 (53.12, 100.00) 8.60 (0.00, 81.24)	59.38 (50.00, 82.82) 89.84 (78.12, 100.00) 38.68 (9.42, 81.24)	57.82 (49.45, 85.94) 59.53 (53.12, 85.94) 1.40 (0.00, 7.77)	1.92 <0.001 0.002
**G** **Week 0** **Week 5** **Percent of improvement**	66.67 (25.96, 88.88) 77.78 (37.04, 100.00) 18.15 (0.00, 156.78)	62.97 (88.88, 25.96) 96.30 (66.66, 100.00) 38.92 (8.35, 156.78)	66.67 (25.16, 85.18) 72.22 (37.04, 88.88) 4.67 (0.00, 42.68)	1.92 <0.001 0.01
**WB** **Week 0** **Week 5** **Percent of improvement**	77.78 (37.04, 100.00) 96.00 (76.00, 100.00) 1.02 (0.00, 36.11)	87.00 (72.00, 98.00) 99.00 (96.00, 100.00) 12.68 (2.04, 36.11)	94.00 (76.00, 98.00) 94.00 (76.00, 98.00) 0.00 (0.00, 0.00)	0.44 <0.001 <0.001
**PE** **Week 0** **Week 5** **Percent of improvement**	80.56 (75.00, 100.00) 90.83 (75.00, 100.00) 0.00 (0.00, 25.92)	80.56 (75.00, 100.00) 94.44 (91.66, 100.00) 17.25 (0.00, 25.92)	80.00 (75.00, 90.00) 80,62 (75.41, 90.00) 0.00 (0.00, 0.00)	1.16 <0.001 0.002

Experimental group: mCIMT: modified constraint-induced movement therapy; control group: UTWC: unimanual therapy without containment. MQ: movement quality; DM: dissociated movement; GR: grasp and release; WB: weight bearing; PE: protective extension. Results expressed in medians (IQR, interquartile range: Q1, Q3). Statistical significance when *p* value is < 0.05 (α correction in Mann–Whitney U-test).

## Supplementary Materials

The following are available online at https://www.mdpi.com/2077-0383/9/9/2992/s1, Figure S1: Percent of improvement Shuee evaluation variables, Figure S2: Percent of improvement QUEST scale variables.

## Appendix A

**Table A1:** Designed task applied in the intervention protocol. Examples of the designed tasks applied in the five week intervention protocol for both therapies (mCIMT and UTWC).

Intervention Weeks for Both Groups (mCIMT and UTWC)	Designed Tasks for the First Hour (Examples)	Designed Task for the Second Hour (Examples)
**-Week 1-** **Shoulder Flexion/Shoulder external rotation**	1. Shoe boxes attached on the table. First one, then two and up to three stacked boxes where the parents will place light objects (such as crumpled papers, small boxes or a ball), which the child will first pull and then take. 2. The parents will give the child a small and light ball, which he/she will try to throw higher and higher or towards a target. 3. The parents will throw balloons or bubbles, which the child will try to hit with his/her hand.	1. The parents will stick plasticine balls on the wall at increasing heights, which the child will try to remove. 2. The parents will put a cardboard or continuous paper stuck on the wall; using finger paint, the child will try to draw a picture or put his/her hand with paint on the paper. 3. The parents will place stickers at different heights on the wall and the child will try to cover them with his/her hand.
**-Week 2-** **Elbow extension**	1. The parents will place a rope from one end of a chair to the other and hang different objects (strings, pieces of paper, deflated balloons...). They will ask the child to try and touch these objects from a sitting position on the floor. 2. The parents will place different tools in front of the affected arm on a table, and the child will perform the elbow extension movement to touch them. 3. The parents will place different objects on top and in front of the child, who will try to reach, touch or take them off.	1. The parents will put pieces of plasticine glued on the table and the child will try to take them off, reproducing the elbow extension movement. 2. The child will glue small empty bottles, with marbles or other elements inside, on the table. Then, he/she will try to knock them down. 3. The child will use the magnet board placed in front of him/her.
**-Week 3-** **Forearm supination**	1. The parents will put stickers on the palm of the hand or on the forearm of the affected arm. 2. The parents will place a light object on the palm of the affected hand (for example, a colored pompom) and the child will keep it for a time. 3. The child will comb, play a trumpet or noisemaker, etc.	1. The child will take things stuck under the table. 2. The child must remove rings towards supination 3. The child will use the affected hand to remove objects stuck on his/her t-shirt (at the level of the abdomen).
**-Week 4-** **Wrist extension**	1. The child must roll a ball, bottle. 2. The child must hit a piano or a drum placed vertically. 3. The parents will place an object on the back of the hand and the child will try to raise the back of his/her hand towards the extension while keeping the object from falling.	1. The child will remove pieces fallen from the wall. 2. The parents will push cardboard boxes or other elements and the child will try to throw them off the table. 3. The child will smash packing paper, balls and/or soft objects with the palm of his/her hand.
**-Week 5-** **Grasp**	1. The child will grasp, hold and transfer light and long objects. The child will grasp, hold and transfer heavy, long and rough objects. 2. The child will grasp, hold and transfer rough, light and spherical objects. The child will grasp, hold and transfer rough, heavy and spherical objects. 3. The child will grasp, hold and transfer smooth, light and long objects. The child will grasp, hold and transfer smooth, heavy and long objects.	1. The child will grasp, hold and transfer smooth, light and spherical objects. The child will grasp, hold and transfer smooth, heavy and spherical objects. 2. The child will grasp, hold and transfer small, light, long and rough objects. The child will grasp, hold and transfer small, heavy, long and rough objects. 3. The child will grasp, hold and transfer small, rough and spherical objects. The child will grasp, hold and transfer small, rough, heavy and spherical objects.
