# Supplementary material for: Unimanual Intensive Therapy with or without Unaffected Hand Containment in Children with Hemiplegia. A Randomized Controlled Pilot Study
Source: J Clin Med. 2020 Sep 16;9(9):2992. doi: 10.3390/jcm9092992 (PMC7563985; doi:10.3390/jcm9092992)
Supplement: Supplementary file 1 [file jcm-09-02992-s001.pdf]

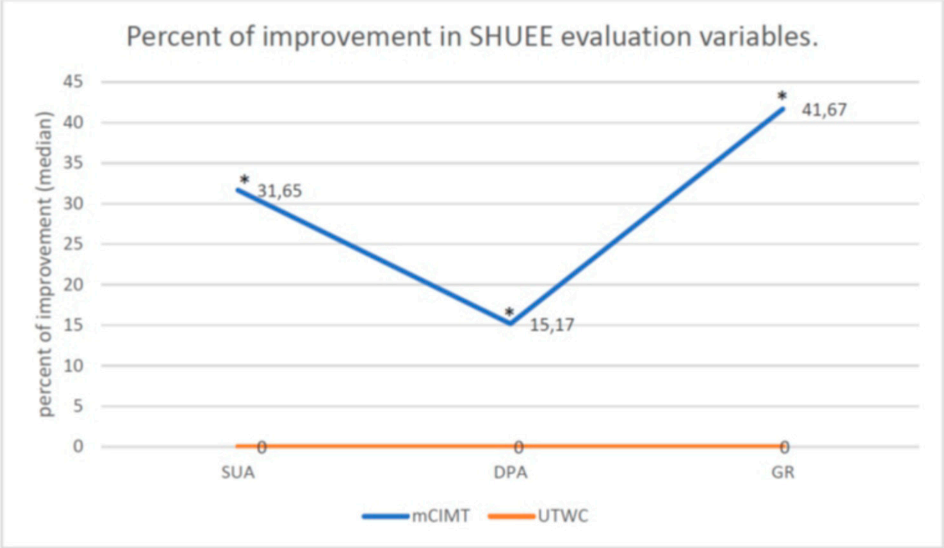

Figure S1. Percent of improvement in Shuee evaluation variables: SUA: Spontaneous Use Analysis; DPA: Dynamic Positional Analysis and GR: Grasp-Release. In this image is observed the great increase in the percent of improvement after applying the protocol in group mCIMT and not changes are seen in the UTWC group. *Statistically significant when p value < 0.05.

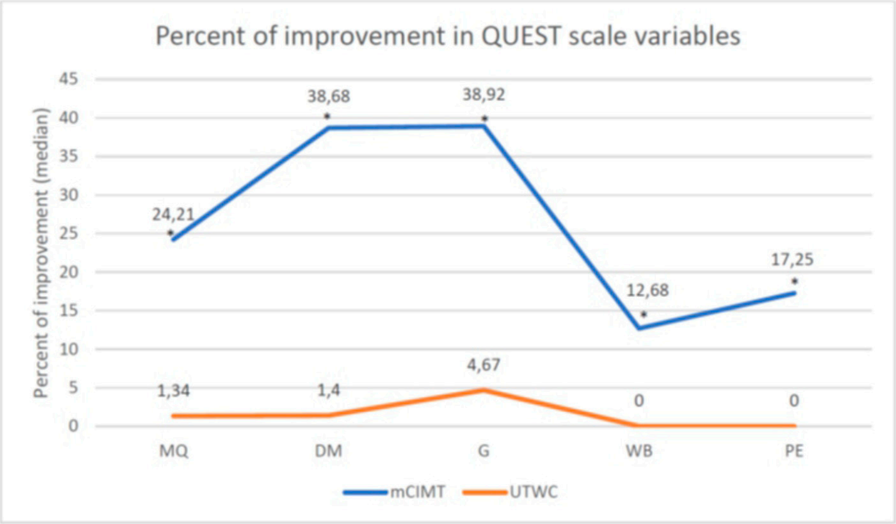

Figure S2. Percent of improvement in QUEST scale variables: MQ: Movement Quality; DM: Dissociated Movements; G: Grasp; WB: Weight Bearing; PE: Protective Extension. In this image is observed the great increase in the percent of improvement after applying the protocol in group mCIMT and small changes are seen in the UTWC group (MQ, DM, G). *Statistically significant when p value < 0.05.
